# Current status of robot-assisted surgery implementation in endometriosis centers: an international multicentric cross-sectional study

**DOI:** 10.1007/s00404-025-08081-9

**Published:** 2025-06-12

**Authors:** Harald Krentel, Nicolas Samartzis, Dimitrios Rafail Kalaitzopoulos, Alin Stefan Constantin, Karl-Werner Schweppe, Julian Matthias Metzler, Dimitrios Andrikos, Isabell Witzel, Rudy Leon De Wilde, Jörg Keckstein, Laurin Burla

**Affiliations:** 1 Department of Obstetrics, Gynecology and Gynecological Oncology, Bethesda Hospital, Duisburg, Germany; 2 Department of Gynecology and Obstetrics, Hospital Schaffhausen, Schaffhausen, Switzerland; 3 Department of Gynecology and Obstetrics, Saarland University Hospital, Homburg, Germany; 4 Stiftung Endometriose-Forschung, Westerstede, Germany; 5 Department of Gynecology, University Hospital of Zurich, Zurich, Switzerland; 6 Clinic of Gynecology, Obstetrics and Gynecological Oncology, University Hospital for Gynecology, Pius-Hospital Oldenburg, Medical Campus University of Oldenburg, Oldenburg, Germany; 7 Endometriosis Clinic Dres. Keckstein, Villach, Austria

**Keywords:** Endometriosis, Deep endometriosis, Endometriosis surgery, Robotic surgery, Robot-assisted surgery, Robotic-assisted surgery, RAS, Robot-assisted laparoscopy, RAL, Minimally invasive surgery, MIS, MIGS, Endometriosis centers

## Abstract

**Purpose:**

The surgical treatment of endometriosis, which is routinely performed by minimally invasive approach, is developing towards an increasing complexity in deep endometriosis. While RAS appears to be gaining importance, there are few real-life data on its use for patients with endometriosis. The aim of this study is to investigate the current use of RAS in certified endometriosis centers in Central Europe.

**Methods:**

In this international multicentric cross-sectional study, an online branching survey was sent to certified endometriosis centers in Austria, the Czech Republic, Germany, and Switzerland. This survey contained 47 questions including proportion of use, indications, advantages and barriers, technical aspects, and training in RAS.

**Results:**

Of the 97 centers contacted, 66% (*n* = 64) participated. RAS is used for the treatment of endometriosis in 60.8% (*n* = 31) of the centers with access to a SR, which corresponds to 48.4% of all participating centers. In Austria, 81.8% (*n* = 9) of centers have SR access, respectively, 88.9% (*n* = 8) use RAS for endometriosis; in Switzerland, 91.6% (*n* = 11) and 36.4% (*n* = 4); and in Germany, 74.4% (*n* = 29) and 62.1% (*n* = 18). The reported advantages of RAS include precision (80%, *n* = 40), instrument mobility (74%, *n* = 37), and visualization (72%, *n* = 36). Compared to CLS, RAS is preferred in multidisciplinary cases (84.6%, *n* = 22), and overweight patients (61.5%, *n* = 16) and deep endometriosis (61.5%, *n* = 16). Specific anatomical indications for RAS vs. CLS include FU (57.7%, *n* = 15), C (53.9%, *n* = 14), and FB (50%, *n* = 13) (#Enzian classification). Patient outcomes of RAS compared to CLS are rated as advantageous in 69.2% (*n* = 18). The main barriers for RAS for centers without an SR include costs (100%, *n* = 12) and lack of scientific evidence (33.3%, *n* = 4). 69.2% (*n* = 18) have dedicated robotic teams, 42.3% (*n* = 11) have a second console, 69.2% (*n* = 18) have a simulator, and 34.6% (*n* = 9) have training programs. A total of 65.4% (*n* = 17) believes that RAS will replace CLS in selected cases, and 73.1% (*n* = 19) would prefer RAS if costs were equal.

**Conclusion:**

This study demonstrates that RAS is already being used in approximately half of the participating endometriosis centers. While the proportion of RAS procedures compared to CLS is increasing, it still remains comparatively low. Country-specific differences in the use of RAS are evident and are most likely linked to healthcare system structures. Participating centers report both technical and general surgical advantages, as well as specific benefits in cases of deep endometriosis. The main barriers include costs and a lack of scientific evidence. Further research is needed to evaluate the long-term role of RAS in the management of endometriosis.

## What does this study add to the clinical work

**Table:** 

This study provides the first comprehensive overview of real-world use of RAS for endometriosis in certified centers across Central Europe. It highlights current advantages and barriers, addresses technical and training-related aspects, and identifies specific indications where RAS appears to be favored over CLS. In doing so, it supports clinical decision-making and fosters informed engagement with RAS in the treatment of endometriosis.	

## Introduction

Endometriosis, defined as the presence of endometrium-like glandular and stromal cells outside the uterine cavity, affects about 10% of women of reproductive age [1]. Three main forms are distinguished: superficial peritoneal endometriosis, ovarian endometriosis, and deep endometriosis [2]. Endometriosis can lead to dysmenorrhea, dyspareunia, dysuria, dyschezia, infertility, and non-cyclic pelvic pain, significantly impacting the quality of life of affected individuals, which in turn has relevant consequences for healthcare and socioeconomic systems [3, 4].

Endocrine therapy is evidence-based, effective, and a safe option recommended for the treatment of endometriosis-associated pain [5–7]. The pharmacological options suppress the symptoms but do not eliminate the local manifestations of the disease. Therefore, surgical removal of endometriosis lesions remains a cornerstone of endometriosis therapy, particularly when medication is insufficient, in cases of infertility, or in cases of severe organ involvement, like bowel or ureteral disease.

Especially deep endometriosis represents a surgical challenge as the lesions often invade multiple pelvic structures and distort the anatomy [8]. Diagnosis and treatment of endometriosis are becoming increasingly systematic, comprehensive, and, where indicated, multidisciplinary. Imaging-based preoperative staging, classification and treatment planification are the current gold standard of care [9]. The goal is to minimize inadequate surgeries, reduce the number of surgeries and ideally aiming for one precise and efficient surgery with the appropriate expertise [7, 9–11].

MIS has become the standard approach in deep endometriosis surgery [7]. In general, it is associated with less pain, a shorter hospital stays, and fewer complications [12]. A further technical development of CLS within the spectrum of MIS is RAS. RAS has its origins in research funded by NASA and the US military in the 1970 s, which led to the first Da Vinci systems by Intuitive Surgical (Sunnyvale, California, USA) in the early 2000s. Over the following years, these systems gained widespread adoption in various surgical specialties. In recent years, a variety of new surgical robots have been developed by various companies, targeting a wide range of applications [13].

Recently, RAS also gained traction in the surgical management of deep endometriosis. The role of RAS, with strengths, such as advancement and optimization of MIS, precise dissection, and improved visualization, as well as drawbacks like cost considerations, is actively debated within the medical community. As often the case with new developments, the data remains inconsistent. [14–25]. The current state of the literature suggests that RAS is non-inferior to CLS in the treatment of deep endometriosis. The outcomes are comparable, with no clear evidence of increased complication rates or perioperative morbidity [26–28].

However, there are still few data on how RAS is used in real-life clinical practice. This study aims to fill this research gap by analyzing the current status of RAS in certified endometriosis centers in Central Europe with regard to various aspects, such as extent of use, advantages, barriers, technical considerations, and surgical training.

## Materials and methods

### Endometriosis centers

On behalf of the Scientific Endometriosis Foundation (SEF) and the European Endometriosis League (EEL), we contacted German-speaking certified endometriosis centers in Austria, the Czech Republic, Germany, and Switzerland. Inclusion criteria were that the centers perform endometriosis surgeries and certification by EuroEndoCert (EuroEndoCert GmbH, Mannheim, Germany).

### Survey

In February 2024, a literature review was conducted to identify literature on RAS for endometriosis as well as similar surveys in other surgical fields. Based on these findings, H.K. and L.B. developed the questionnaire. Following this, a pilot phase took place where the questionnaire was sent to co-authors (N.S., D.R.K., A.S.C., K.-W.S.), all gynecologists with several years to decades of experience in endometriosis care, for corrections and adjustments.

The result was a branching survey in German with 47 questions, which were divided into demographic aspects of the participants/participating centers, general implementation of RAS with questions about application areas, financial aspects, advantages, barriers, and specifically the implementation of RAS in endometriosis with questions about case numbers, indications, technical aspects, surgical team, training and education, and prospects. The branching survey consisted of following consecutive sections: whether the clinic has its own RAS system (or accesses one at another clinic); whether it is used for gynecology; and whether it is specifically used for endometriosis surgery. Depending on what applied, the survey ended earlier or later for each center. For example, the questions on barriers to RAS were only answered by those who do not yet have access to an SR. The general advantages were assessed by all centers with an SR access. The endometriosis-specific RAS questions were only answered by those centers that actually perform RAS for endometriosis, only these centers had the opportunity to complete the survey until the end.

Regarding the questions about surgical teams, curriculum, and training, comparing training levels across countries can be challenging due to variations in titles. To address this, we tried to simplify the terminology to enhance clarity and facilitate comparisons. A fellow is a physician who has completed residency training and is employed to pursue further specialization in a specific area, such as endometriosis or RAS. A specialist is a physician who has successfully passed a board examination, such as in gynecology and obstetrics. A consultant is typically a specialist who works independently within the clinic, similar to a senior or attending physician, referred to in German as "Oberärztin/-arzt". A senior consultant is a position one level above a consultant, serving as an intermediary role between a consultant and a chief physician. This role might include responsibilities such as heading a section or serving as a deputy head, referred to in German as "Leitende/r Ärztin/Arzt, Leitende/r Oberärztin/Oberarzt".

For simplicity, the term "center" is used to refer to endometriosis centers in general. Specific distinctions regarding hospital type are explicitly stated when relevant.

The questionnaire was created using an online tool (SurveyMonkey, San Mateo, CA, USA) and was sent via email in March 2024, including an information letter and a note stating that this is a scientific assessment. The centers were individually contacted by the SEF (K.-W.S.), and participation was limited to a one-time submission via the provided link. As stated in the information letter, the goal was for a senior medical member from each center to complete the survey. After two months, a reminder was sent, and the survey was closed after four months. The survey was intentionally conducted anonymously to encourage honest feedback.

### Statistical analysis

Statistical analysis was performed with IBM SPSS Statistics 27 (Endicott, NY, USA). Summary statistics for categorical variables were presented as numbers and percentages, while continuous variables were expressed as median along with the corresponding IQR.

## Results

### Response rate and participating endometriosis centers

**Fig. 1:**
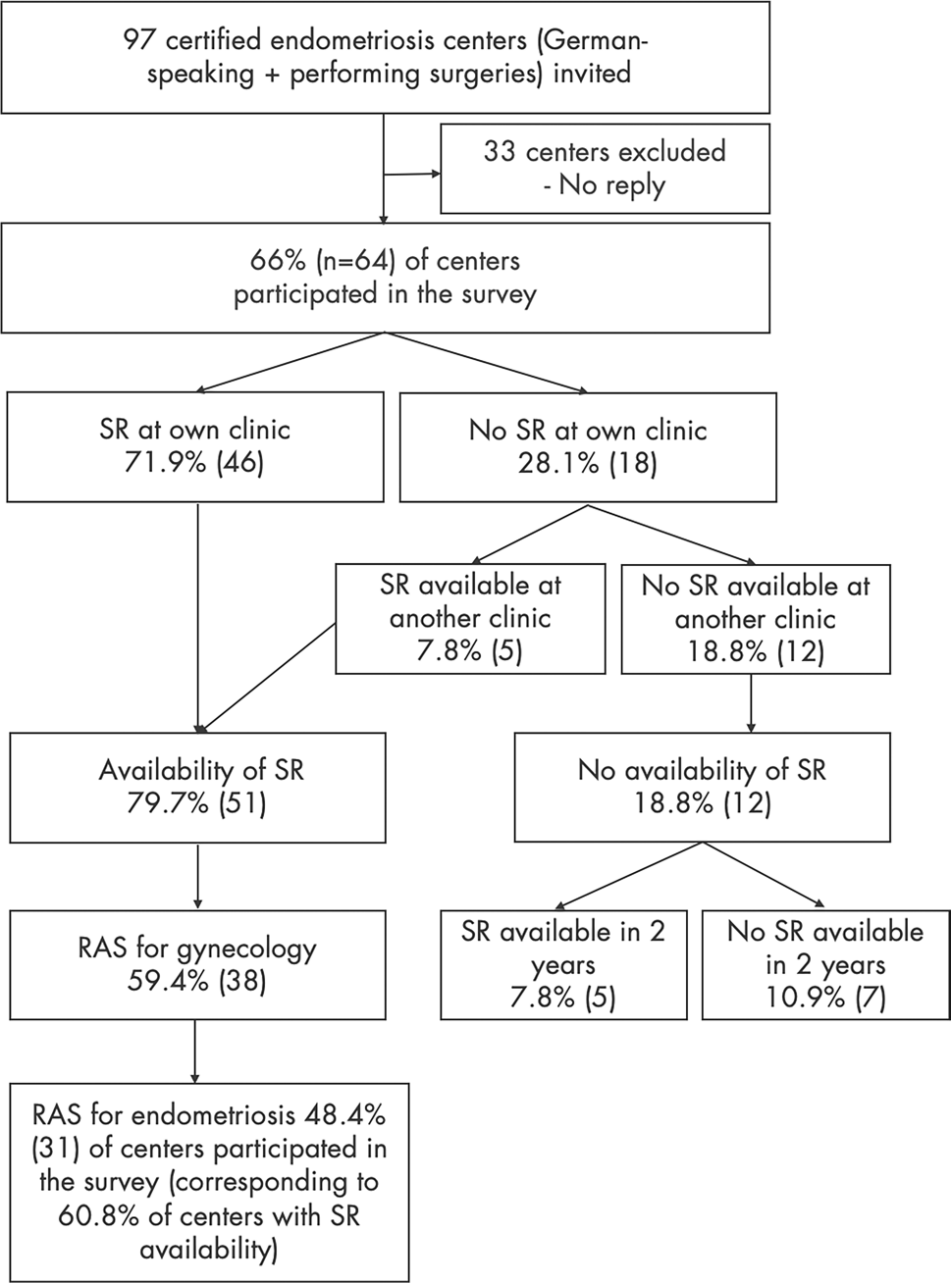
This flowchart illustrates the structure of the branching survey and the process from inviting the centers to participation. The topics of the subgroups, such as availability of a surgical robot (SR) or robot-assisted surgery (RAS) for endometriosis, were then further elaborated with more specific questions within the subgroups. The term 'SR at own clinic 'refers to the entire clinic, not just one department

Of the 97 centers contacted, 66% (*n* = 64) participated. Numbers and percentages presented in the following sections vary by topic in relation to the total number of responding centers. This is primarily due to the structure of the branching survey, as the questions were directed to different numbers of centers (see section *Survey* in *Material and Methods*). Further deviations in the number of responses within certain questions are due to varying response rates. However, the proportion of centers that answered the respective questions assigned to them was high, with an average response rate of 88.3%. Figure 1 contains a flowchart illustrating both the study participation of the centers and the structure of the branching survey.

60.9% (*n* = 39) of the participating centers are based in Germany, 18.8% (*n* = 12) in Switzerland, 17.2% (*n* = 11) in Austria, and 3.1% (*n* = 2) in the Czech Republic. In 38.7% (*n* = 24) of the centers, the questionnaire was completed by a chief physician/head of department and in 61.3% (*n* = 38) by a consultant or senior consultant. Among the responding centers, 52.4% (*n* = 33) were university hospitals, 28.6% (*n* = 18) were non-academic tertiary centers, and 19.1% (*n* = 12) were regional hospitals. There were no private clinics or private practices participating.

### General implementation of RAS

71.9% (*n* = 46) of the 64 centers have their own SR. 7.8% (*n* = 5) reported having access to an SR at another center when needed. The distribution of RAS among the participating centers can be found in Fig. 1.

In 94% (*n* = 47) of centers with access to an SR, it is used in colorectal surgery and urology. In 76% (*n* = 38) of centers with access to an SR, and therefore in 59.4% (*n = 38*) of all participating centers, RAS is used for gynecological surgeries in general.

The SR is purchased in 66% (*n* = 33), financed through leasing in 10% (*n* = 5), and rented in 2% (*n* = 1). 84% (*n* = 42) of respondents indicate that RAS is more expensive than CLS, while the remaining 16% (*n* = 8) are unaware of the costs.

When deciding between RAS and CLS for gynecological surgery, factors considered include case complexity (78%, *n* = 39), offering newer/more advanced treatment options (26%, *n* = 13), multidisciplinary considerations (e.g., colleague expertise in CLS, such as in urology, general surgery) (62%, *n* = 31), and surgical costs (26%, *n* = 13). Additionally, 12% (*n* = 6) mentioned other aspects, such as capacity/access to the SR, time efficiency, faster and more comfortable surgery, management decisions limiting gynecology access to RAS due to costs. In 28% (*n* = 14), the center imposes restrictions on case selection for RAS due to financial considerations.

**Fig. 2:**
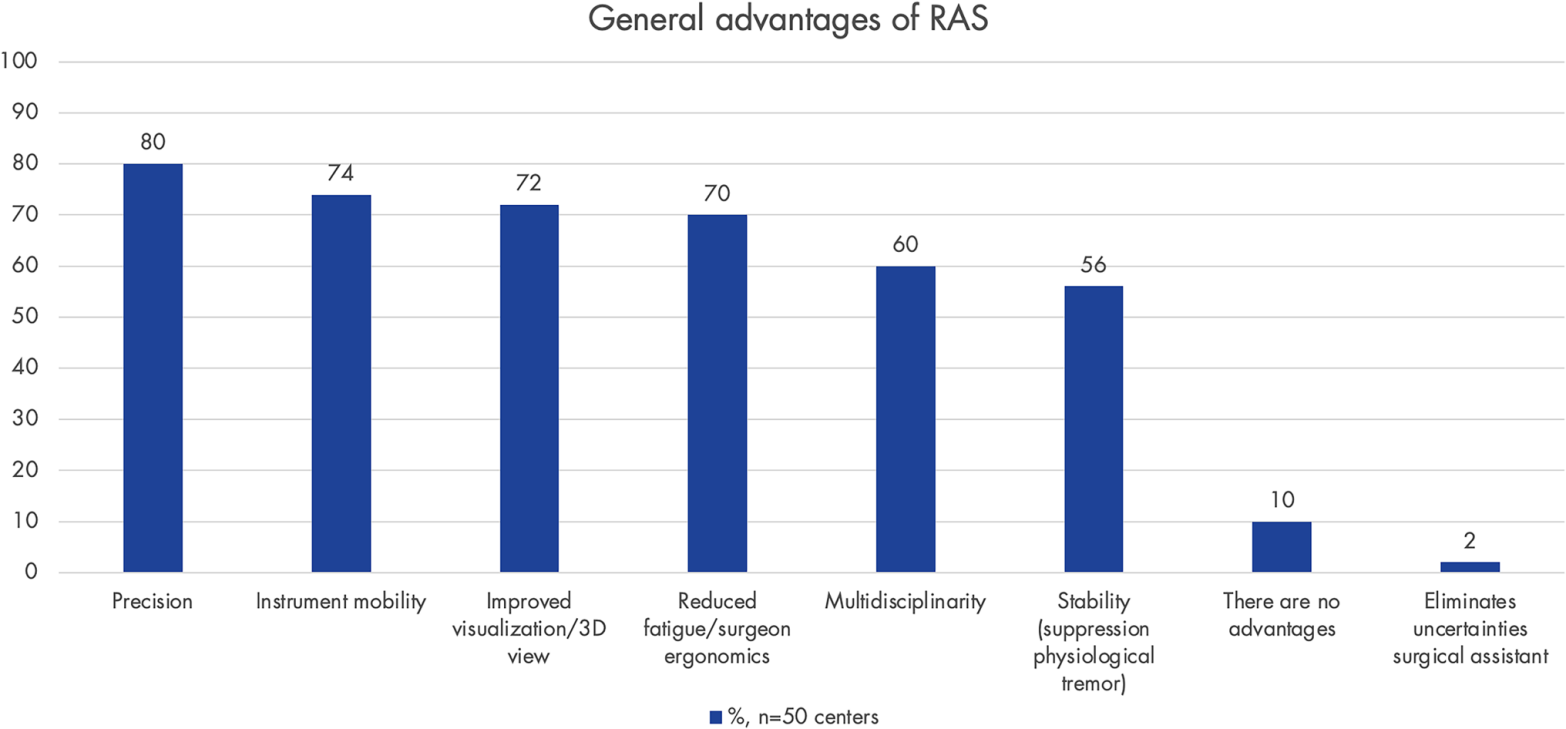
This shows where the participating centers generally see the surgical advantages of robot-assisted surgery (RAS). Only centers with access to an SR were asked (*n* = 51); response rate: 98% (*n* = 50)

**Fig. 3:**
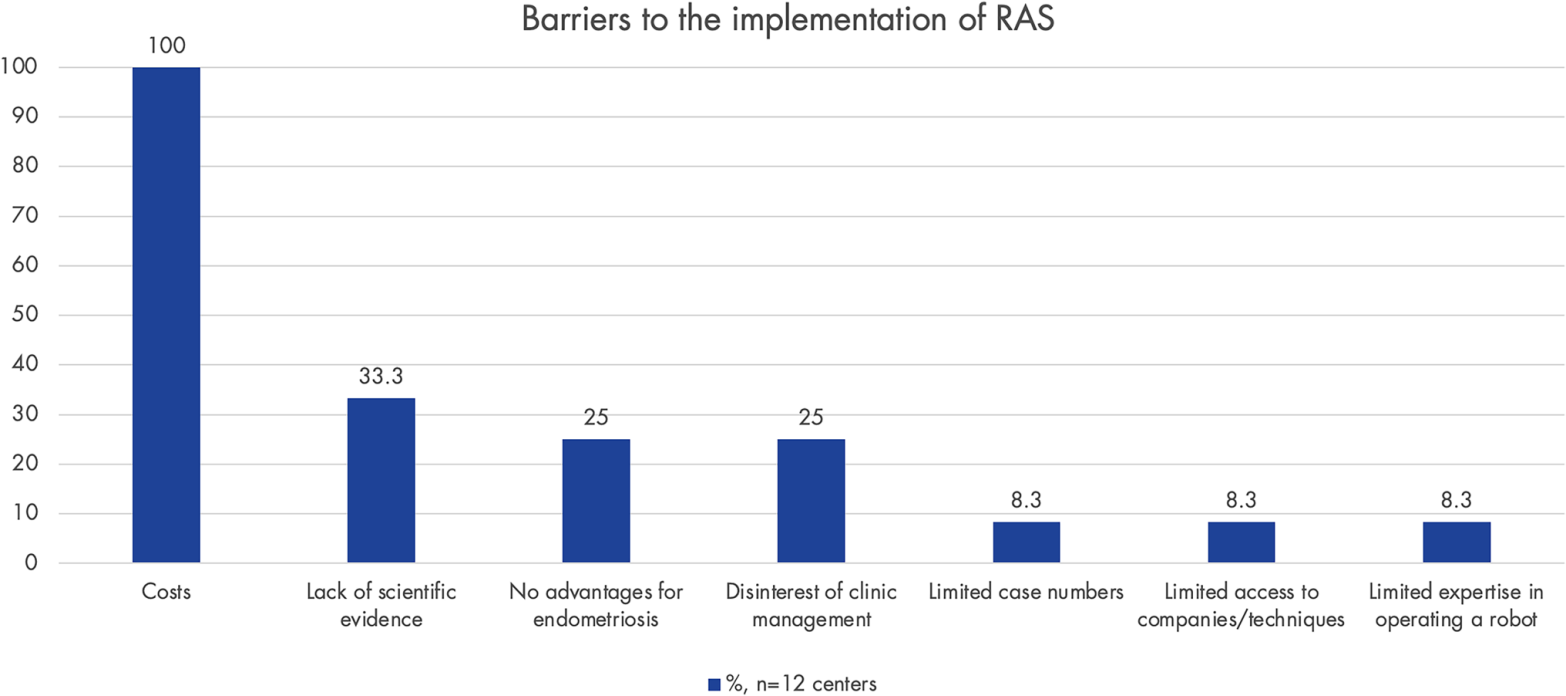
Perceived barriers to implementing robot-assisted surgery (RAS) from the centers that do not yet have surgical robots available (*n* = 12 centers). Only centers without access to a SR were asked (*n* = 13); response rate: 92.3% (*n* = 12)

Figure 2 illustrates the general advantages of RAS as reported. The barriers to implementing RAS from the perspective of centers without an SR are shown in Fig. 3.

### Implementation of RAS for Endometriosis

In 60.8% (*n* = 31) of the centers with availability of an SR (or 48% when considering all 64 participating endometriosis centers), RAS is used for endometriosis cases (Fig. 1).

In 53.9% (*n* = 14) of these centers, the SR is available for endometriosis cases one day/week, in 38.5% (*n* = 10) it is available for two days/week, and in 7.7% (*n* = 2), it is available for three days/week.

**Fig. 4:**
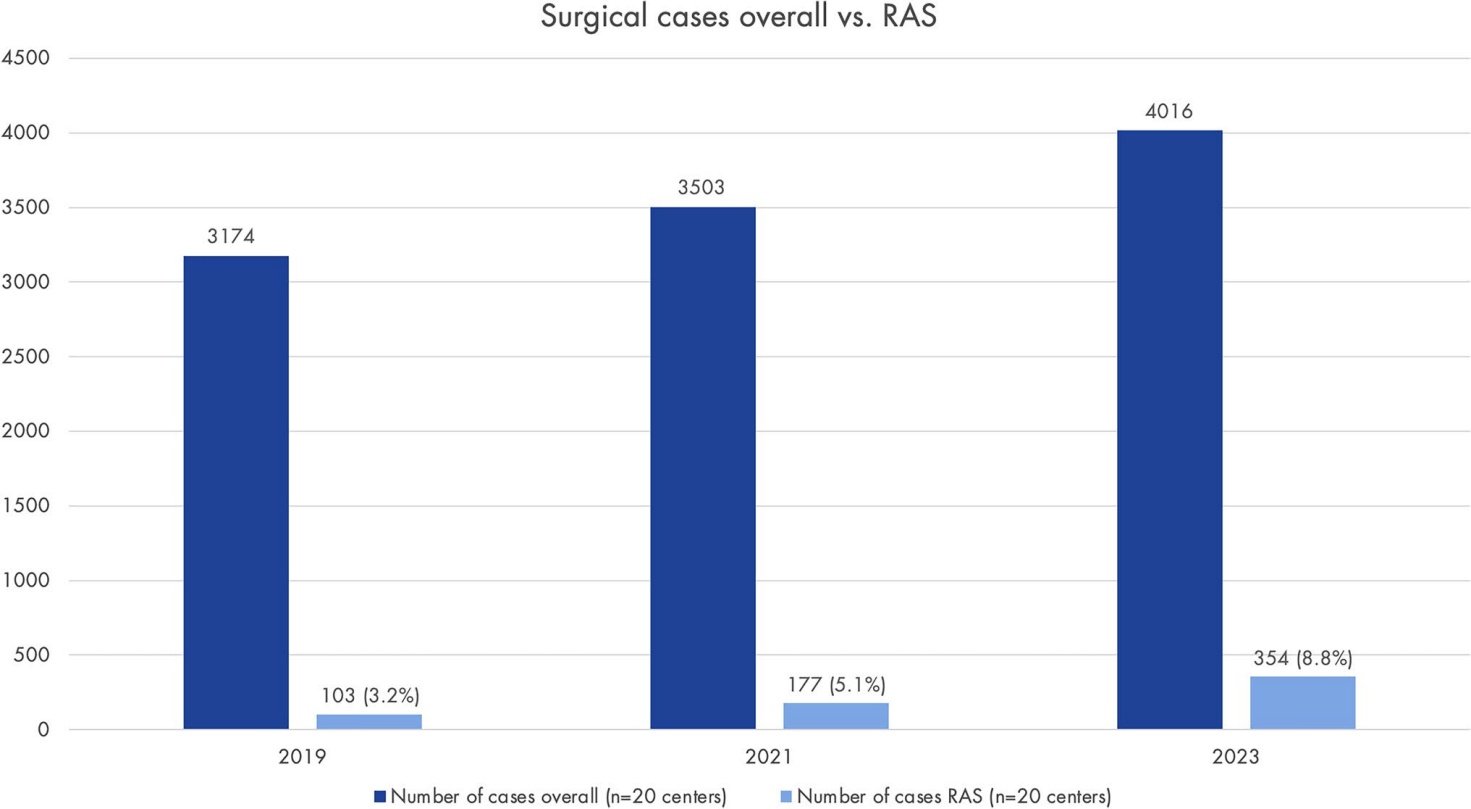
This shows the total number of surgical endometriosis cases (regardless of surgical technique, MIS/open) in 2019, 2021, and 2023 compared to the proportion of robot-assisted surgery (RAS) cases (data from 20 endometriosis centers). Only centers using RAS for endometriosis were asked (*n* = 31); response rate with data for all three years: 64.5% (*n* = 20)

The centers have a median of 3.5 years (IQR 1.1–4.8) of experience in RAS for endometriosis. In 76.9% (*n* = 20) of cases, the chief physicians perform RAS for endometriosis, additionally in 84.6% (*n* = 22) senior consultants, in 61.5% (*n* = 16) consultantss, in 11.5% (*n* = 3) fellows, and in 3.6% (*n* = 1) residents. In all centers where RAS is used for endometriosis cases, it is also used in other gynecological areas (urogynecology, oncology). Figure 4 shows the overall number of endometriosis surgeries vs. RAS in 20 of the participating centers in 2019, 2021, and 2023.

**Fig. 5:**
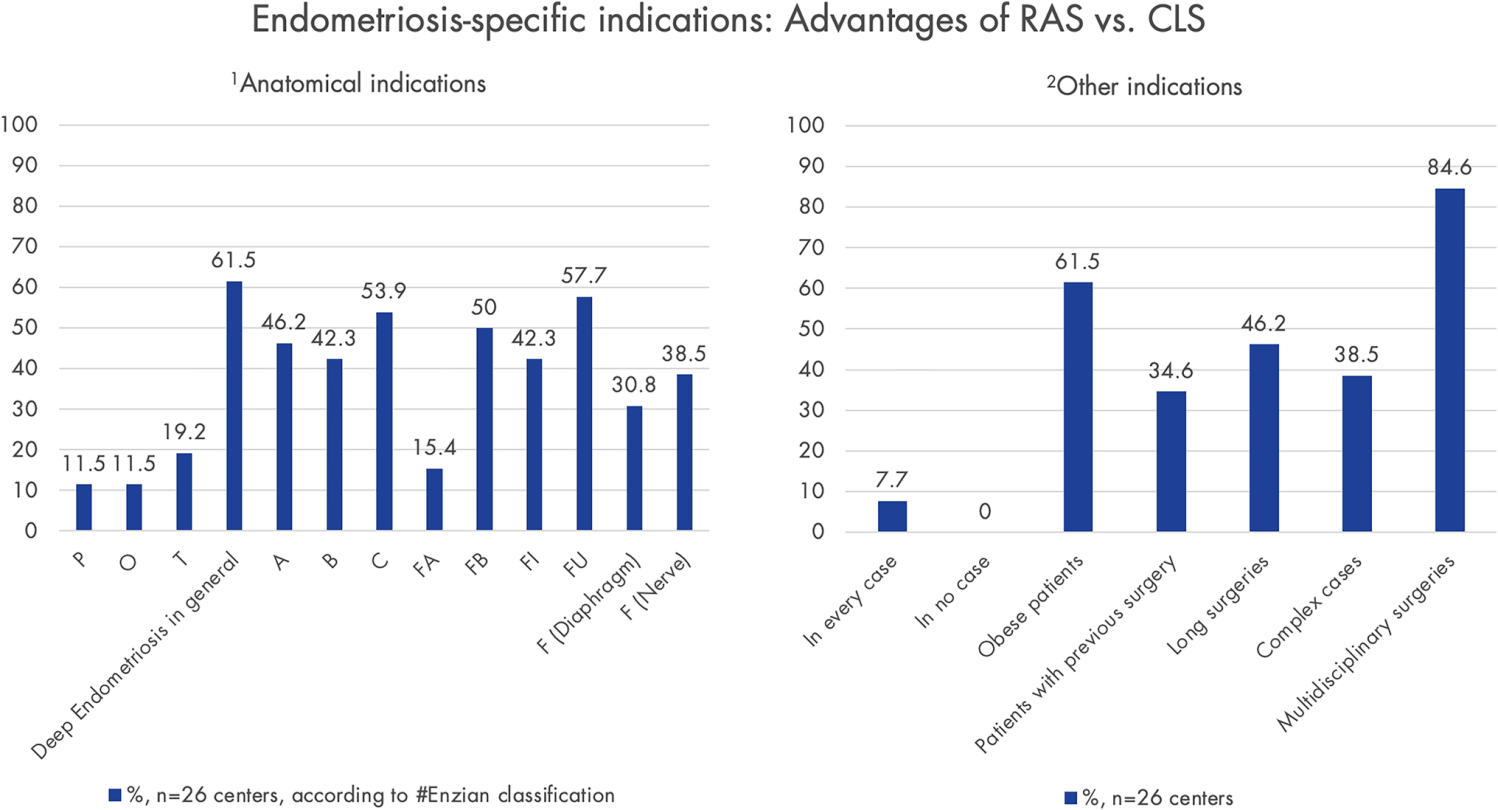
Extent to which robot-assisted surgery (RAS) is considered advantageous compared to conventional laparoscopic surgery (CLS) for endometriosis-specific indications (anatomical localization according to the #Enzian classification [29]. Only centers using RAS for endometriosis were asked (*n* = 31); response rate: 83.4% (*n* = 26)

The respondents consider RAS advantageous over CLS in multidisciplinary cases, obese patients and deep endometriosis. In regard of anatomical indications, RAS is considered advantageous vs. CLS in C and FU (#Enzian classification) (Fig. 5). In 92.3% (*n* = 24) of the centers, there are collaborations with other specialties (e.g., urology, colorectal surgery) for combined RAS procedures.

**Fig. 6:**
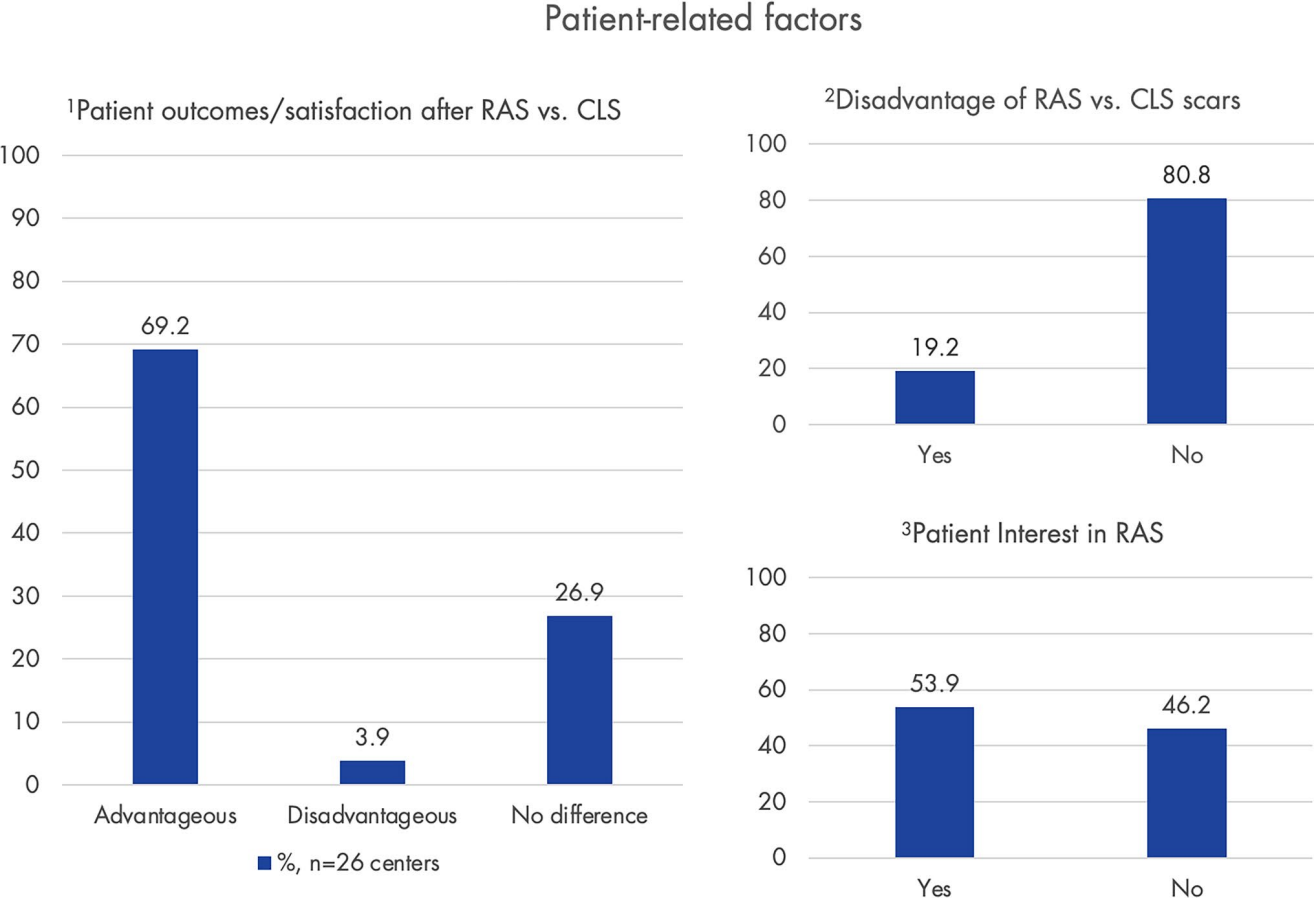
Here certain patient-related factors as reported by the 26 centers are shown. Graph 1 illustrates how the centers generally assess patient outcomes/satisfaction following robot-assisted surgery (RAS) compared to conventional laparoscopic surgery (CLS). Graph 2 shows whether patients have perceived scars from RAS as disadvantageous (usually higher scars in RAS than CLS). Graph 3 indicates whether patients are interested in RAS for endometriosis/whether they inquire about it on their own. Only centers using RAS for endometriosis were asked (*n* = 31); response rate: 83.4% (*n* = 26)

Patient-related factors, as reported by the centers, are shown in Fig. 6. In approximately 70% (*n* = 26) of participating centers, RAS has been rated as advantageous over CLS in terms of observed patient outcomes/satisfaction.

### Country and hospital-type specific differences

In Switzerland, 91.6% (*n* = 11) of the participating centers have access to an SR, followed by 81.8% (*n* = 9) in Austria and 74.4% (*n* = 29) in Germany. Among these, SRs are most frequently used for the treatment of endometriosis in Austria, with 88.9% (*n* = 8) of centers, followed by 62.1% (*n* = 18) in Germany and 36.4% (*n* = 4) in Switzerland.

Regarding differences based on hospital type, SR are most found in academic centers, with 90.9% (*n* = 30), followed by non-academic tertiary centers with 61.1% (*n* = 11), and regional hospitals with 33.3% (*n* = 4). Of these, SR are used for endometriosis in 76.7% (*n* = 23) of academic centers, 40% (*n* = 6) of non-academic tertiary centers, and 50% (*n* = 2) of regional hospitals.

### Technical aspects

**Fig. 7:**
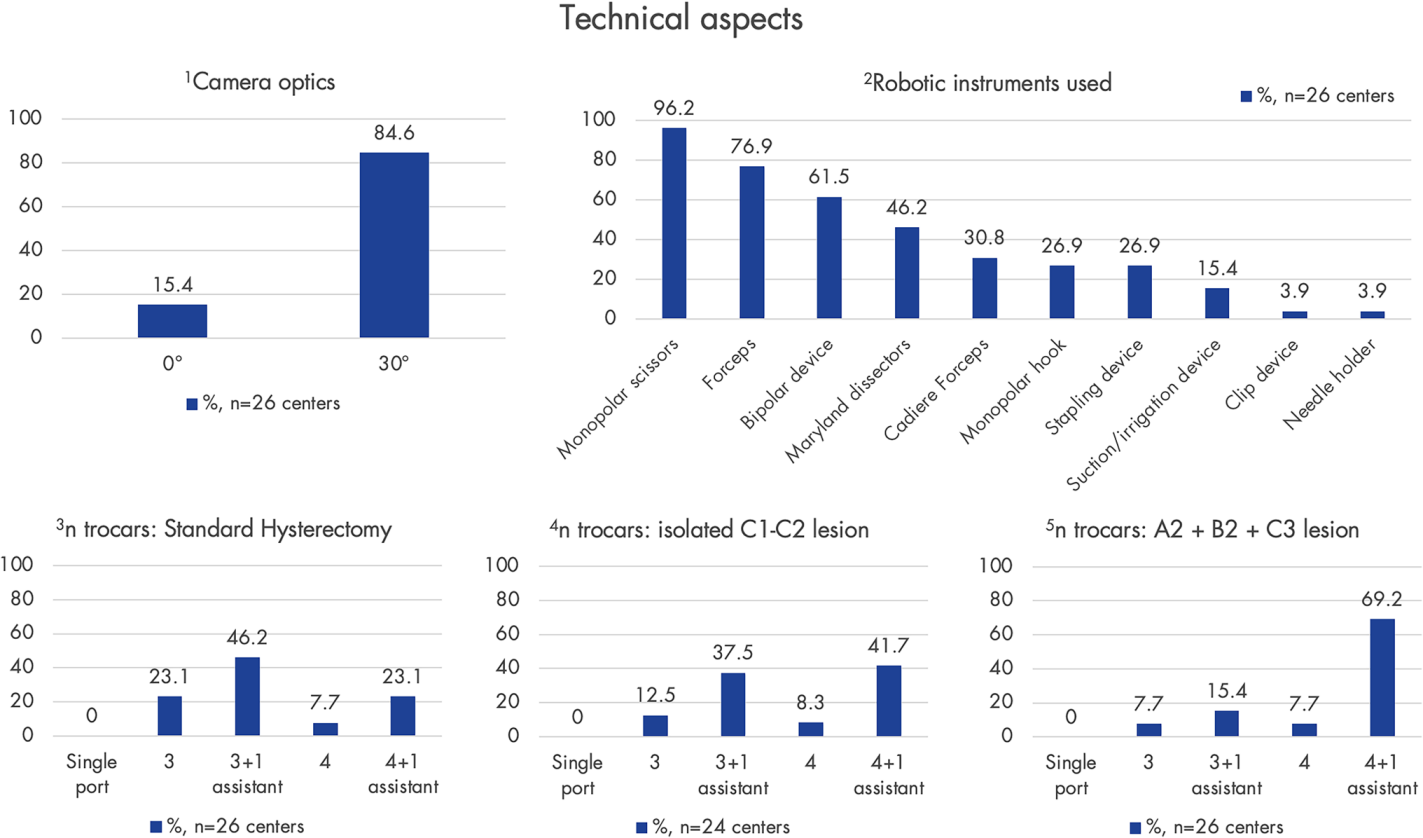
An overview of technical aspects, as indicated by the centers, is provided here. Graphs 1 and 2 show the type of optics used and commonly used instruments. Graphs 3–5 display the number of trocars for certain representative robot-assisted surgery (RAS) procedures (according to the #Enzian classification). Only centers using RAS for endometriosis were asked (*n* = 31); response rate: 83.4%/77.4% (*n* = 26/*n* = 24)

The currently most prevalent SRs are those from Intuitive Surgical (Sunnyvale, CA, USA) (Da Vinci Xi (69.2%, *n* = 18), DaVinci X (23.1%, *n* = 6), DaVinci Si (7.7%, *n* = 2)). Other systems available in the clinics include ROSA® (Zimmer Biomet, Warsaw, IN, USA) in 3.9% (*n* = 1), Senhance® (Asensus Surgical, Durham, NC, USA) in 7.7% (*n* = 2), and the Versius Surgical System (CMR Surgical, Cambridge, UK) in 3.9% (*n* = 1). Further findings regarding technical aspects are shown in Fig. 7.

### Surgical teams, training, and research

**Fig. 8:**
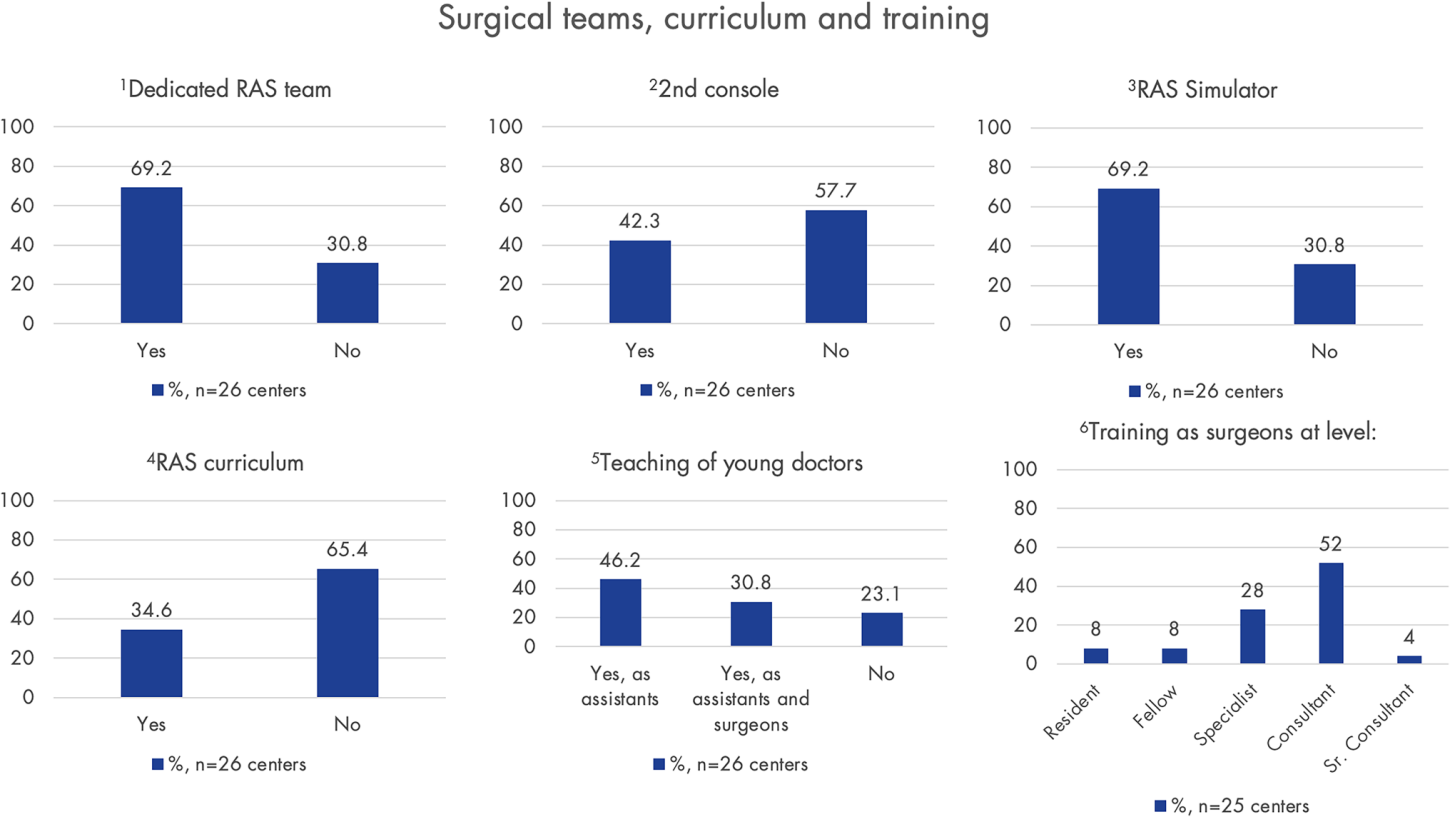
This section presents various aspects related to surgical teams, curriculum, and training in robot-assisted surgery (RAS). Graph 1 shows how many centers report having a dedicated RAS team, including nurses, assistants, and surgeons. Graphs 2 and 3 illustrate the prevalence of a second console and simulators, which are essential for training. Graph 4 indicates how prevalent dedicated RAS curricula are used to train junior robotic surgeons, graph 5 shows whether young doctors are trained in RAS, and graph 6 displays the level at which training to become a robotic surgeon begins. Only centers using RAS for endometriosis were asked (*n* = 31); response rate: 83.4%/80.6% (*n* = 26/*n* = 25)

If training as a surgical assistant is provided, it occurs in 50% (*n* = 12) at the level of a resident, in 29.2% (*n* = 7) at the specialist level, in 16.7% (*n* = 4) at the consultant level, and in 4.2% (*n* = 1) at the fellow level. Figure 8 shows when surgical training begins, as well as findings on surgical teams, specific internal RAS curricula, and training measures.

**Fig. 9:**
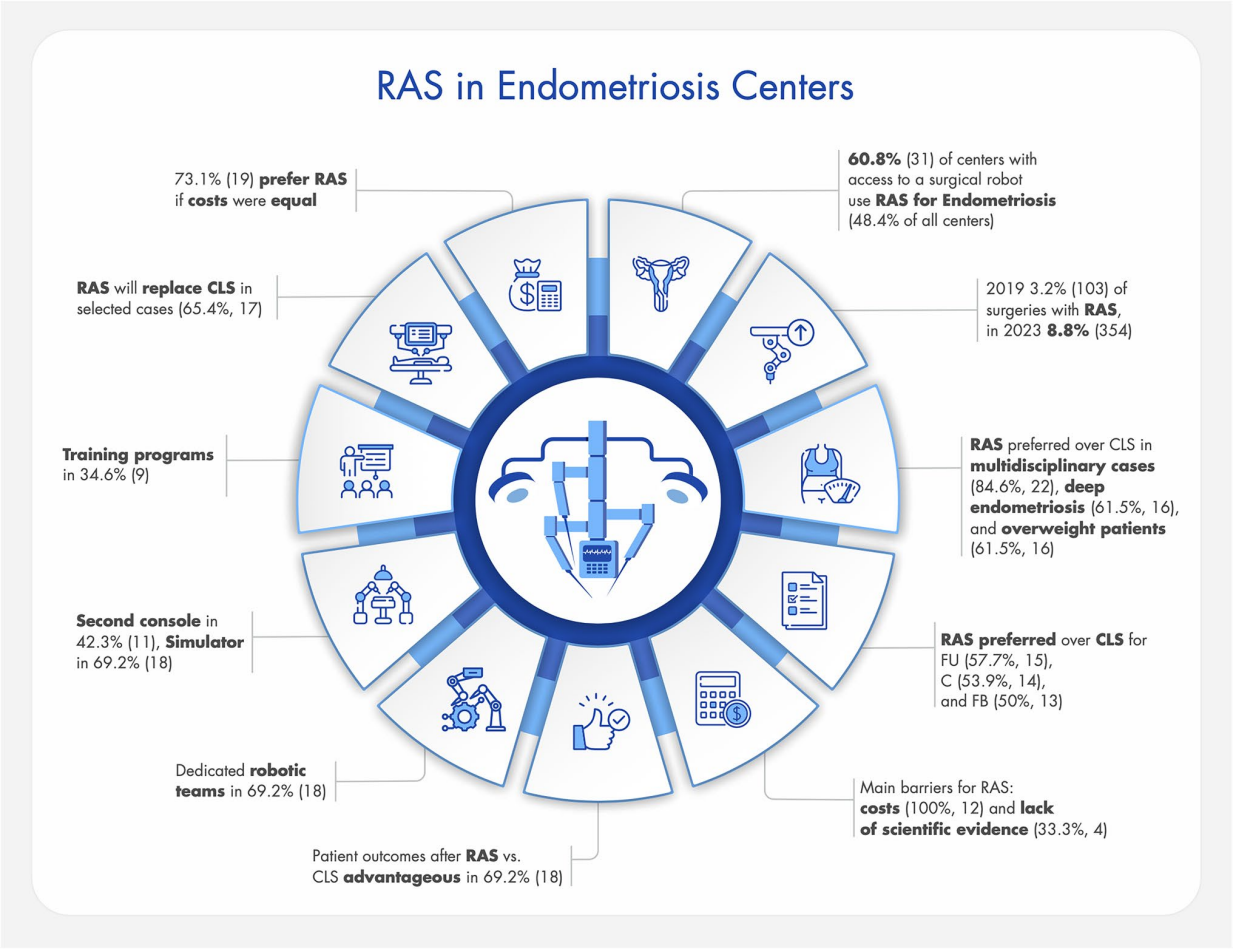
presents key findings from the study including % and the corresponding number of participating centers (robot-assisted surgery (RAS), conventional laparoscopic surgery (CLS), according to the #Enzian classification)

In 53.9% (*n* = 14) of the centers, research on RAS for endometriosis is being conducted. In 92% (*n* = 23) of the centers, further research on RAS for endometriosis is considered valuable. In 34.6% (*n* = 9), the centers have specific protocols for RAS and/or follow-up. In 65.4% (*n* = 17), the quality of RAS at the center is monitored and ensured in some way (e.g., database, patient registry) (Fig. 9).

### Future perspectives

65.4% (*n* = 17) of the respondents state that RAS will replace CLS for endometriosis in selected cases, 3.9% (*n* = 1) state, it will replace it in all cases, and 30.8% (*n* = 8) do not expect CLS to be replaced. However, if the costs were the same, 73.1% (*n* = 19) would prefer RAS for MIS for endometriosis.

## Discussion

This is, to the best of our knowledge, the first international multicenter analysis of the use of RAS in the treatment of endometriosis, providing practical insights into its application. Although the use of RAS for the treatment of endometriosis is still relatively new, 48.4% of all participating centers and 60.8% (*n* = 31) of centers with access to an SR in this study, reported utilizing RAS for endometriosis.

Interestingly, national differences are evident: 91.6% (*n* = 11) of Swiss centers have SR access, but only 36.4% (*n* = 4) use RAS for endometriosis, compared to Austria with 81.8% (*n* = 9) and 88.9% (*n* = 8), and Germany with 74.4% (*n* = 29) and 62.1% (*n* = 18). These differences are likely due to variations in healthcare system structures. Austria seems to have a more centralized system with a combination of centers for the treatment of endometriosis and RAS centers. This might be the result of the Austrian national health plan, whereas Switzerland operates a federal system, with its 26 cantons independently organizing their healthcare systems [30, 31]. Although SRs are widely available in Switzerland and Germany, their use in the treatment of endometriosis still seems to be limited.

Compared to the overall number of endometriosis surgeries in the responding centers, the number of RAS is still very low. However, the data collected in this study indicates an upward trend, with the use of RAS in endometriosis nearly tripling from 3.2% in 2019 to 8.8% in 2023. In our opinion, RAS should be available in those centers managing a high percentage of complex deep endometriosis cases. This would also be in line with the results of this study. For innovative techniques like RAS, centralization is crucial to ensure sufficient case volumes for clinical practice, patient safety and research. This is also reflected in hospital types. SRs are most found in academic centers (90.9%, *n* = 30), of which 76.7% (*n* = 23) use RAS for endometriosis.

Although the current scientific evidence regarding RAS for endometriosis remains sparse, with limited data on safety, efficacy, and cost-effectiveness [28], most of the studies show that RAS is not inferior to CLS in terms of intra- and perioperative complications, blood loss, conversion rates, and re-hospitalization rates and its use appears particularly promising for complex cases of deep endometriosis [14, 16–21, 27, 32]. This is in line with the results of this study, as the respondents consider RAS advantageous over CLS in cases of deep endometriosis, especially with involvement of the ureters (#Enzian FU) and the rectum (#Enzian C). The fact that involvement of diaphragm and pelvic nerves has been evaluated with a lower rate, might be related to the limited number of centers managing these special types of symptomatic deep endometriosis.

Additionally, the responding centers rated RAS advantageous over CLS in multidisciplinary surgeries and in obese patients. The multidisciplinary approach as an advantage is evident as other specialists, such as urologists, increasingly perform MIS almost exclusively with RAS [19]. Regarding obese patients, data on endometriosis is sparse, but advantages have been demonstrated in related fields [33–36].

The multidisciplinary presurgical preparation of complex endometriosis allows for the optimal positioning of the patient, number of trocars and robotic arms, placement of trocars, instrumentation for all involved disciplines, making time-consuming intraoperative changes unnecessary. Commonly mentioned general advantages of RAS included precision, instrument mobility, improved visibility/3D visualization, reduced fatigue/improved ergonomics for the surgeon [37], multidisciplinarity, and stability (suppression of physiological tremors). These general advantages of RAS align with the current literature [38, 39].

In 69.2% of centers, patient outcomes and satisfaction following RAS are generally perceived as advantageous compared to CLS. Interestingly, in 80.8% (*n* = 21) of centers, the more cranially located scars in RAS compared to CLS are not considered a disadvantage. Furthermore, newer approaches in RAS, such as single-port access or Robot-Assisted Vaginal Natural Orifice Transluminal Endoscopic Surgery (RvNOTES), could further enhance cosmetic outcomes [40, 41].

However, RAS currently seems to be disadvantageous compared to CLS in areas, such as costs, operation duration, and hospitalization time [14, 17, 18, 20, 42]. It is a well-known challenge when introducing new techniques that they are often compared to the work of experienced experts with extensive proficiency in established methods. As routine increases, RAS and CLS will ultimately become more comparable. New studies suggest that RAS offers advantages in certain outcomes, such as reduced postoperative voiding dysfunction after colorectal endometriosis surgery. Additionally, shorter hospital stays have been observed with RAS compared to CLS [43, 44]. The previously cited disadvantages of RAS appear to be less definitive as expertise grows and caseloads increase. A look at urology shows that around 20 years ago, RAS was still met with skepticism, with an estimated 10% of radical prostatectomies in the U.S. being performed using RAS [45]. Today, RAS is almost indispensable in urological surgery and dominates in radical prostatectomies [46, 47].

The main barrier for the implementation of RAS appears to be its costs. Among respondents using RAS, 84% (*n* = 42) reported being aware of the costs at their centers, noting that they are higher for RAS compared to CLS. Nevertheless, only 28% (*n* = 14) of centers currently face restrictions from hospital administration regarding which procedures can be performed using RAS. Costs are also actively discussed in the literature [42, 48]. However, as it is a newer technology and different alternative techniques are entering the market, intensifying competition, it is expected that the cost issue will partially level out in the future [49]. Optimal utilization of robotic systems should be targeted: currently, 94% (*n* = 47) of centers with a SR report that the SR is also used by other disciplines, such as colorectal and urological surgery. It is worth to mention, that especially multidisciplinary multi-organ endometriosis surgeries are usually well-compensated by the health care systems and studies indicate a downward trend in costs with increasing case numbers [50, 51]. Our data show that centers vary the number of robotic arms and auxiliary trocars – and thus the surgical costs – depending on the complexity of the case.

A lack of scientific evidence, no perceived advantage of RAS over CLS, and disinterest from hospital management are additional reasons why RAS has not yet been implemented in some of the centers.

Currently, 53.9% (*n* = 14) of participating centers state that they are conducting research on RAS in endometriosis, and 92% (*n* = 23) believe that further research in this field is beneficial. Additionally, 65.4% (*n* = 17) of centers have internal quality assurance for RAS cases through databases or patient registries. Larger studies, especially to prove the perceived advantages of RAS over CLS are needed and currently underway [52].

Our data shows that currently more than half of participating centers do not have a specialized RAS curriculum or a second console. Without doubt, there is a need for specialized training programs to improve expertise in RAS, especially for the next generation of robotic surgeons, as in most of the centers, RAS is being used on consultant level only. The availability of simulators and mentoring programs for staff training is crucial as the level of experience and training influence the outcomes of RAS.

We see the strengths of this study in being the first international and multicentric assessment on the use of RAS in endometriosis surgery conducted among certified endometriosis centers. The questionnaire was designed as a branching survey, so that only centers indicating access to an SR or using of RAS for endometriosis were prompted to answer the respective questions. In this way, we aimed to gather insights that are as relevant as possible and based on current expertise.

A limitation of this study is that it is based on an online survey. Although we cannot guarantee that all responses fully reflect the actual circumstances within the respective centers, particularly since the data were provided by a single representative per center, the survey was conducted anonymously to encourage honest and open responses. We assume that the participating centers, which are committed to certification and the treatment of endometriosis patients, provided the most accurate information possible. A bias cannot be excluded as it is possible that centers already using RAS were more likely to participate. Nevertheless, we consider the participation of 64 certified endometriosis centers to offer a relatively representative snapshot. Due to the design of the study, it was not possible to collect data at the individual patient or surgeon level (including complication rates or patient-reported outcomes). This may affect the clinical translatability of the findings and should be further investigated. A further important area for future research lies in the investigation of cost-related barriers, including a comparative cost analysis between RAS and CLS, as this is a critical factor for health policy decision-makers. Another challenge lies in the presentation of the data. Since we conducted a branching survey, only certain proportions answered specific questions. The response rate within each question is another factor that led to varying numbers of participants per item. However, the average response rate across questions was high, at 88.3%. Overall, designing and structuring such a survey is complex, and the outcome difficult to predict. If all centers, regardless of their expertise, were asked to answer every section, the data would have lower validity.

In conclusion, this study demonstrates that while RAS is still relatively new in the field of endometriosis, it is already being utilized in approximately half of the participating endometriosis centers. The percentage of RAS procedures has tripled over just a few years, though the overall proportion still remains comparatively low. Country-specific differences in RAS use are evident, likely reflecting healthcare system structures, such as centralization. Participating centers report both technical and general surgical advantages of RAS, as well as specific benefits for certain indications, particularly in cases of deep endometriosis. Further research, along with increasing experience and analyses of cost/benefit evaluations, is essential to assess the long-term role of RAS in the management of endometriosis.

## Data Availability

Data are available upon reasonable request. No datasets were generated or analysed during the current study.

## Abbreviations

RAS: Robot-assisted surgery
SR: Surgical robot
CLS: Conventional laparoscopic surgery
MIS: Minimally invasive surgery
IQR: Interquartile range
